# Combination Therapy of Cisplatin and Naturally Induced Exosomes from Treated HeLa Cells: A Novel Drug-Free Strategy for Cervical Cancer Treatment

**DOI:** 10.34172/apb.46428

**Published:** 2026-06-15

**Authors:** Sanaz Aghajani, Nasim Vousooghi, Abbas Hajifathali, Iman Seyhoun, Javad Verdi

**Affiliations:** ^1^Department of Applied Cell Sciences, School of Advanced Technologies in Medicine, Tehran University of Medical Sciences, Tehran, Iran; ^2^Research Center for Cognitive and Behavioral Sciences, Tehran University of Medical Sciences, Tehran, Iran; ^3^Hematopoietic Stem Cell Research Center, Shahid Beheshti University of Medical Sciences, Tehran, Iran; ^4^Gene, Cell & Tissue Research Institute, Tehran University of Medical Sciences, Tehran, Iran

**Keywords:** Cervical cancer, Apoptosis, Exosome, Targeted therapy, Effect delivery, Exosome delivery

## Abstract

**Purpose:**

Cisplatin and several other chemotherapeutic agents remain central to the treatment of cervical cancer, yet their therapeutic benefits are frequently constrained by significant systemic toxicity. Exosomes—small extracellular vesicles released by various cell types—are increasingly examined as potential carriers for drug delivery. Despite this interest, it is still not well understood whether these vesicles can independently propagate the biological effects triggered by a drug, even when the drug itself is no longer present. In this study, we explored whether exosomes collected from cisplatin treated HeLa cells could transfer apoptotic signals to untreated HeLa cells, a phenomenon we refer to as "Effect Delivery."

**Methods:**

Exosomes released by cisplatin treated HeLa cells were isolated through ultracentrifugation and subsequently characterized using standard analytical methods. To determine the appropriate working concentration, the cytotoxic impact of these exosomes on recipient cells was examined through the MTT assay. The ability of the exosomes to trigger apoptosis was then evaluated using flow cytometry, along with quantitative real time PCR (qRT PCR) to measure changes in the expression of key apoptosis related genes.

**Results:**

Exosomes isolated from cisplatin treated HeLa cells were found to induce apoptosis in 57.6% of recipient HeLa cells. This response was accompanied by upregulation of pro apoptotic genes, including BAX, Caspase 3, TP53, and TNF α, along with downregulation of antiapoptotic genes such as BCL2 and BIRC5. Notably, this pro apoptotic effect appeared to be selective for malignant cells, as no comparable apoptotic response was detected in noncancerous HEK293 cells.

**Conclusion:**

Our findings indicate that exosomes released from drug treated cancer cells can convey apoptotic signals to neighboring malignant cells, even in the absence of direct drug transfer. This observation points to a possible drug independent approach for amplifying antitumor responses and offers new perspectives on the role of exosome mediated communication in cancer therapy.

## Introduction

 One of the most prevalent cancers affecting women worldwide is cervical cancer. In 2022, the World Health Organization (WHO) reported that cervical cancer was the fourth most common cancer among women, after lung, colorectal, and breast cancers, with over 660,000 new cases and about 350,000 deaths.^1,2^ Chronic infections with high-risk strains of the human papillomavirus (HPV), specifically HPV-16 and HPV-18, are responsible for more than 95% of these cases. Low- and middle-income nations have disproportionately higher incidence and mortality rates, mostly as a result of restricted access to efficient screening programs and HPV vaccinations.^3-5^

 Currently, the treatment of cervical cancer includes surgery, radiation therapy, and chemotherapy. Cisplatin is one of the chemotherapy agents used due to its strong antitumor effects. However, its significant adverse effects, such as nephrotoxicity, peripheral neuropathy, ototoxicity, myelosuppression, and gastrointestinal toxicity, usually complicate its therapeutic use. These adverse effects can considerably affect the quality of life of patients and limit the therapeutic dose; therefore, there is a need for more targeted and safer treatment approaches.^6-8^

 In recent years, exosomes have emerged as one of the most promising nanocarriers for targeted drug delivery. Secreted by nearly all kinds of cells, these extracellular vesicles can transport chemicals like proteins, mRNA, miRNA, and chemotherapeutics between cells. Intercellular communication, as well as control of apoptosis, depends on them.^9,10^ For use in cancer therapy, many studies have investigated drug-loaded exosomes as delivery vehicles; however, the topic of exosomes naturally secreted by drug-treated cells has remained largely uninvestigated.

 This research introduces a novel strategy by utilizing exosomes derived from HeLa cells pre-treated with cisplatin. We hypothesize that exosomes released from cisplatin-treated HeLa cells contain stress-associated bioactive cargo, such as specific microRNAs, pro-apoptotic proteins, and signaling molecules linked to the DNA damage response and inflammatory pathways. These components may be selectively incorporated into exosomes as a consequence of cisplatin-induced cellular stress. Once transferred to untreated recipient cancer cells, they could promote apoptotic signaling by activating both intrinsic (mitochondrial, e.g., the BAX/TP53 axis) and extrinsic (e.g., TNF-α–mediated) pathways. Unlike traditional approaches that depend on artificial drug loading, this method capitalizes on the endogenous therapeutic potential of exosomes to induce selective cytotoxicity.^11,12^ The central objective of this study is to examine whether exosomes produced by drug-treated cells can selectively trigger apoptosis in cancer cells while minimizing systemic toxicity. By investigating this mechanism, we aim to support the advancement of cancer therapies that are both more precise in their targeting and better tolerated by patients.

## Materials and Methods

 All reagents were of analytical grade and used according to the manufacturers’ instructions.

###  Cell Culture

 HeLa and HEK293 cell lines were obtained from the Royan Research Institute (Tehran, Iran)^13^ and maintained in Dulbecco’s Modified Eagle Medium (DMEM; GE Healthcare Life Sciences, Pittsburgh, PA, USA, Cat. No. 22G0583K) supplemented with 10% heat-inactivated fetal bovine serum (FBS; Thermo Fisher Scientific, Gaithersburg, MD, USA, Cat. No.42G9165K), 1% penicillin-streptomycin (Invitrogen, Eugene, OR, USA, Cat. No. 2196734), and 2 mM L-GlutaMAX (Thermo Fisher Scientific, Cat. No. 42G7812K). Cells were incubated at 37 °C in a humidified atmosphere containing 5% CO2. For exosome isolation, cells were treated with predetermined concentration of cisplatin in an FBS-free medium. Conditioned medium (CM) was collected after 72 hours of incubation.

###  MTT Assay

####  Cisplatin Dose-Response Assay

 To determine the optimal dose of cisplatin, HeLa cells were seeded at a density of 5 × 10^3^ cells per well in 96-well plates. After 24 hours, cells were treated with varying concentrations of cisplatin (5, 10, 20, 40, 60, 80, and 100 μM) and incubated for 24, 48, and 72 hours. Cell viability was assessed using a triplicate MTT assay (Sigma Aldrich, St. Louis, MO, USA, Cat. No. SLBT6721). The absorbance was measured at 570 nm using a TECAN ELISA reader (Salzburg, Austria). Based on the results, 7.95 μM for 72 hours was selected as the optimal dose.

####  Exosome Dose-Response Assay

 HeLa cells were cultured in multiple T125 flasks and treated with 7.95μM cisplatin for 72 hours once cell confluence exceeded 90%. Isolated exosomes from treated cells were applied to HeLa cultures at doses of 50, 100, 150, and 200 μg/mL for 24, 48, and 72 hours. The MTT assay was performed as described, and 166 μg/mL for 72 hours was identified as the effective concentration for further studies.

###  Exosome Isolation

 HeLa cells were progressively adjusted to a serum-free medium over three days to prevent contamination with bovine-derived exosomes, lowering FBS from 10% to 0%. Then, they were treated with 7.95μM cisplatin for 72 hours. The culture supernatant was successively centrifuged at 4 °C: first at 2,000 × g for 20 minutes to eliminate dead cells, then at 10,000 × g for half an hour to get rid of cell waste. At 100,000 x g for 70 minutes, the final supernatant was ultracentrifuged. Re-centrifuged under the same conditions, the pellet was washed with PBS and filtered across a 0.22 μm membrane. The pure exosomes were kept at −80 °C.

####  Adaptation to Serum Free Conditions

 To minimize contamination with exogenous exosomes derived from Fetal Bovine Serum (FBS), HeLa cells were gradually adapted to serum free conditions prior to Cisplatin treatment. Cells were cultured in DMEM with decreasing concentrations of FBS in a stepwise manner over three days (10%, 5%,2%,0%). Each step was maintained for approximately 24 hours to allow cellular adaptation.

 During this period, cell morphology and viability were routinely monitored using light microscopy and trypan blue exclusion assay to ensure minimal stress-induced effects. Only cultures maintaining > 85-90% viability were used for subsequent experiments.

 Control groups were subjected to identical serum-free conditions without Cisplatin treatment to account for any effects induced by serum deprivation alone. This approach reduces potential confounding effects of serum starvation and ensures that observed cellular responses are primarily attributable to Cisplatin treatment and exosome mediated signaling.

###  Exosome Characterization

 Using a BCA assay (DNA biotech, Tehran, Iran, Cat. No. BCA231105), the protein concentration of extracted exosomes was estimated. 1815 μg of protein from 150 mL of conditioned medium from 6–8 × 10^7^ cells produced on average. Transmission electron microscopy examined exosome morphology (TEM) at 80 kV following 2% paraformaldehyde fixation. For scanning electron microscopy (SEM), samples were fixed with 2.7% glutaraldehyde, dehydrated in increasing ethanol concentrations (40% to 98%), and then air-dried. A Nano-ZetaSizer Zeta potential and size distribution were assessed using Malvern Corp., UK; the Zetasizer program (v7.11) was used to analyze data. Western blot study validated the existence of exosomal markers (CD63, CD9, TSG101, HSP70) and the lack of Calnexin.

###  Western Blotting

 RIPA buffer was used to lyse cells, which were then spun at 14,000 rpm for 20 minutes at 4 °C. The Bradford assay was used to determine protein content. Boiling lysers for five minutes with Laemmli buffer, they were then divided using SDS-PAGE. Proteins were moved to Bio-Rad, CA, USA, PVDF membranes (0.2 μm). Membranes were incubated for 1 hour with primary antibodies (anti-CD9, anti-CD63, anti-TSG101, anti-Calnexin, anti-HSP70) (Abcam, Cambridge, UK, Cat. Nos. GR3465124-2, GR3278451-5, GR3342198-1, GR3219047-3, GR3421186-4) following 1 hour of 5% BSA in 0.1% Tween-20 blocking, followed by HRP-conjugated secondary antibodies at 25 degrees C. ECL reagents were used for signal detection. Band density was measured using a Gel Analyzer (v2010a, NIH, USA) and normalized to β-actin.

 Protein concentration was determined using the Bradford assay. To minimize interference from detergents, present in RIPA buffer (including SDS), samples were appropriately diluted prior to measurement. In addition, standard curves were prepared in the same buffer conditions as the samples to ensure accuracy and consistency of the assay. These precautions are commonly employed to mitigate detergent related interference in Bradford based protein quantification. Alternatively, protein quantification was cross validated in selected samples using a detergent compatible assay (BCA).

###  Experimental Design and Treatment Groups

 To further investigate the potential modulatory effect of exosomes on cisplatin response in cancer cells, an additional treatment group (treated Exo/Cis) was included only in HeLa cells. Since HeLa cells represent the malignant model in this study, this group was designed to evaluate whether exosome exposure could influence the cellular response to cisplatin. In contrast, HEK293 cells were used as a non-cancerous reference model primarily to assess baseline cellular responses and cytotoxicity; therefore, this additional treatment group was not included for this cell line.

 To ensure clarity and reproducibility, all experimental groups were defined as follows:

 For HeLa cells, the following groups were included:

1. Untreated control (cells cultured under identical conditions without treatment)
2. Cisplatin-treated group (7.95 µM for 72h)
3. Exosome treated group (cells treated with exosomes derived from Cisplatin-Treated HeLa cells, 166 µg/ml for 72h)
4. Control exosome group (cells treated with exosomes derived from untreated HeLa cells)
5. Combination group (cells treated with both Cisplatin and exosomes)

For HEK293 cells, similar experimental conditions were applied to evaluate the selectivity of the treatment, including:

1. Untreated control
2. Cisplatin-treated group
3. Exosome-treated group (derived from cisplatin-treated HeLa cells)

All treatments were performed under identical culture conditions, and incubation times and concentrations were kept consistent across experiments. Each experiment was conducted in triplicate to ensure reproducibility. This design allowed direct comparison between cancerous and non-cancerous cell responses under identical experimental conditions.

###  Quantitative Real-Time PCR

 Utilizing TRIzol reagent (GeneX, Iran, Cat. No. GXTR240512), total RNA was extracted and measured with a NanoDrop spectrophotometer (Thermo Fisher). Using a SMOBIO cDNA (SMOBIO, Taiwan, Cat. No. SM240315), cDNA synthesis was done. Using BLAST, primers were created and synthesized by SinaClone. Primer sequences are listed in Table 1. qPCR was carried out on an ABI Real-Time PCR System with SYBR Green melting curve analysis, and agarose gel electrophoresis confirmed the specificity of the primer. Expressions were normalized to the B2M reference gene. Table 2 lists the protocols.

**Table 1:** Primers

**Gene**	**Primer**
BIRC5	Forward: CACCCTGGAGCGGATGGC Reverse: CCCCTTGAAGCAGAAGAAACACT
BAX	F: GAGCAGATTATGAAGACAGGGG R: ACGGCGGCAATCATCCTC
BCL2	F: TCGCCCTGTGGATGACTGA R: CAGAGACAGCCAGGAGAAATCA
Caspase3	F: ATCATACAGGGAAGCGAATCAAT R: CGAGATGTCATTCCAGTGCT
P53	F: TACTCCCCTGCCCTCAACA R: GGACCAGACCATCGCTATCT
HSP90	F: TCTGGGTATCGGAAAGCAAGCC R: GTGCACTTCCTCAGGCATCTTG
TNF-ɑ	F: CCCTGACATCTGGAATCTGGAG R: TCAAGGAAGTCTGGAAACATCTGG
PDL1	F: CCAGCACACTGAGAATCAACA R: ATTTGGAGGATGTGCCAGAG
B2M	F: AGATGAGTATGCCTGCCGTGT R: TGCGACATCTTCAAACCTCCAT

**Table 2:** The temperature program of genes

**PCR temperature program**	**Melting curve temperature program**
Step1: 95 °C/ 15min	95 °C 15 sec
Step2: 95 °C/ 15 sec	65 °C
Step3: 60 °C/ 45 sec	Slope: 0.4
Step3*: 62 °C/ 45 sec (BIRC5)	95 °C/ 15 sec
Repeat steps 2-3, 45 times	

###  Apoptosis Analysis by Flow Cytometry

 The BioLegend, USA FITC Annexin V/PI kit (BioLegend, San Diego, CA, USA, Cat. No. B326541) was used to evaluate apoptosis. Cells were stained, rinsed, resuspended in binding buffer (0.5–1.0 × 10^6^ cells/mL), and incubated with 5 μL Annexin V-FITC and 10 μL PI for fifteen minutes in the dark. A FACSCalibur flow cytometer (BD Biosciences, USA) was used to examine samples; data were then processed using the FlowJo program (FlowJo LLC, OR USA).

###  Statistical Analysis

 Triplicate measurements were taken for all experiments; data are shown as mean ± SD. GraphPad Prism 9 (GraphPad Software Inc., La Jolla, CA, USA) was used for statistical analysis; one-way ANOVA followed by Tukey’s post hoc test evaluated statistical importance, with *P* values below 0.05 deemed significant.

## Results and Discussion

###  Determination of Optimal Cisplatin Dose via MTT Assay

 Cells were incubated for 24, 48, and 72 hours and exposed to ascending dosages of cisplatin (5–100 μM) to ascertain the best cisplatin concentration for HeLa cell treatment. The MTT assay was used to evaluate cell viability. In all treatment groups vs the control (*P* < 0.05 to *P* < 0.0001, Figure 1A), a notable, time- and dose-dependent drop in cell viability was noticed.

**Figure 1:**
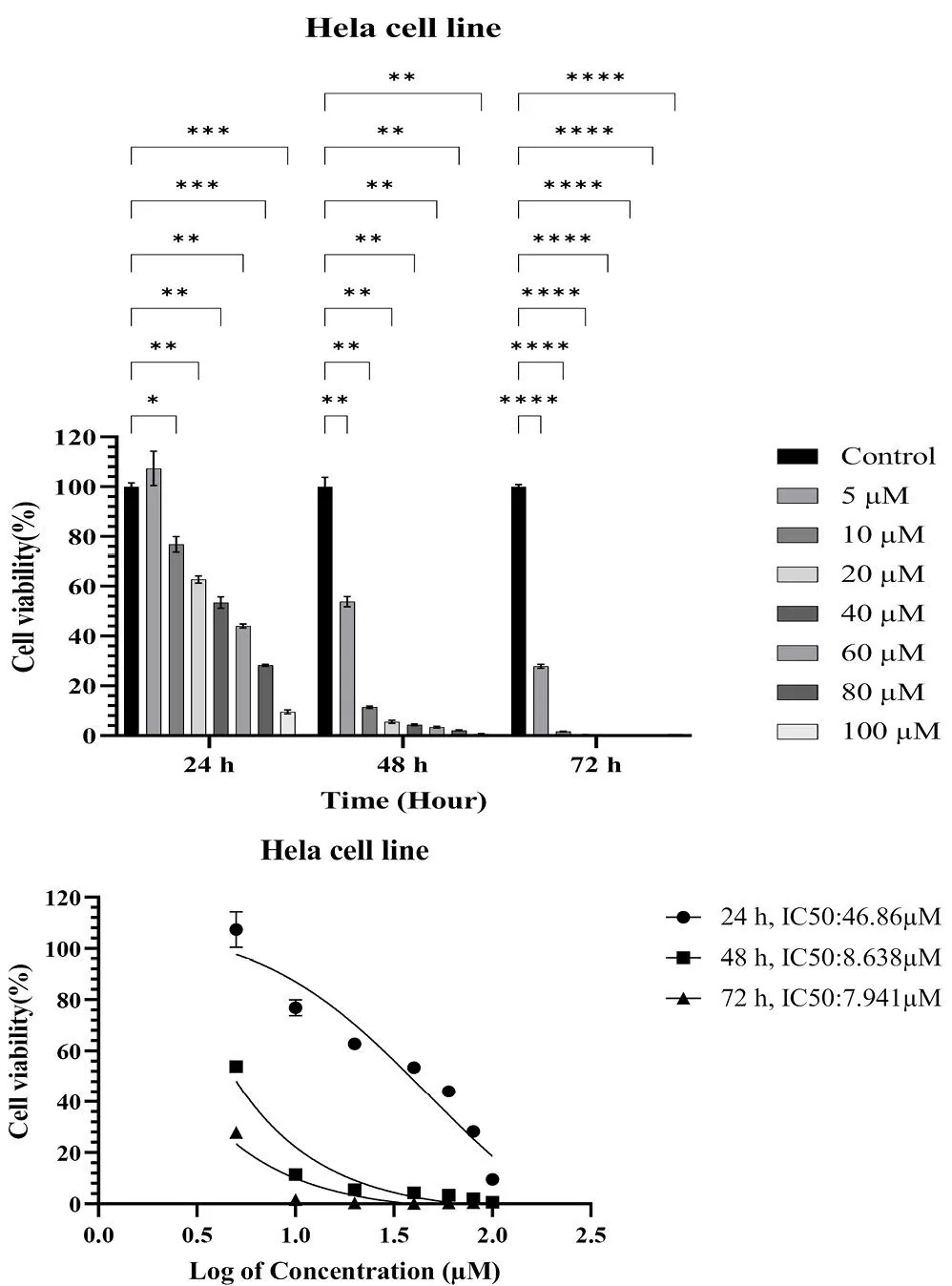
Evaluation of cisplatin cytotoxicity in HeLa cells. (a) Cell viability assessed by MTT assay after exposure to increasing cisplatin doses (5–100 μM) for 24, 48, and 72 h. Data are presented as mean ± SD (n = 3). We performed statistical analysis using one-way ANOVA followed by Tukey’s post hoc test. Significance levels relative to the control group are indicated as *P < 0.05, **P < 0.01, ***P < 0.001, ****P < 0.0001. A dose-dependent trend was observed across all treatment groups. (b) Dose-response curve and calculated IC50 values for cisplatin-treated HeLa cells at different time points.

 Dose-response curve fitting showed that the IC50 values of cisplatin were 46.86 μM at 24 h, 8.64 μM at 48 h, and 7.94 μM at 72 h, respectively (Figure 1B). Considering these findings, the best dosage for more tests—7.95 μM at 72 h—was chosen to balance cytotoxic effectiveness and cell viability.

 Statistical significance relative to control was *P < 0.05 to ****P < 0.0001. A dose-dependent trend was observed across all treatment group.

###  Determination of Exosome Effective Dose via MTT Assay

 An MTT assay was conducted to assess the optimal dosage and treatment duration of exosomes on HeLa cells. HeLa cells were treated with exosomes at doses of 50, 100, 150, and 200 µg/mL and incubated for 24, 48, and 72 hours. An MTT assay was conducted to assess cell viability; statistically significant differences (*P < 0.05 to ****P < 0.0001; Figure 2a) were observed for all treatment groups over time and dose compared to the untreated controls, with notably decreasing cell viability in each treatment group over the 72-hour treatment period.

**Figure 2:**
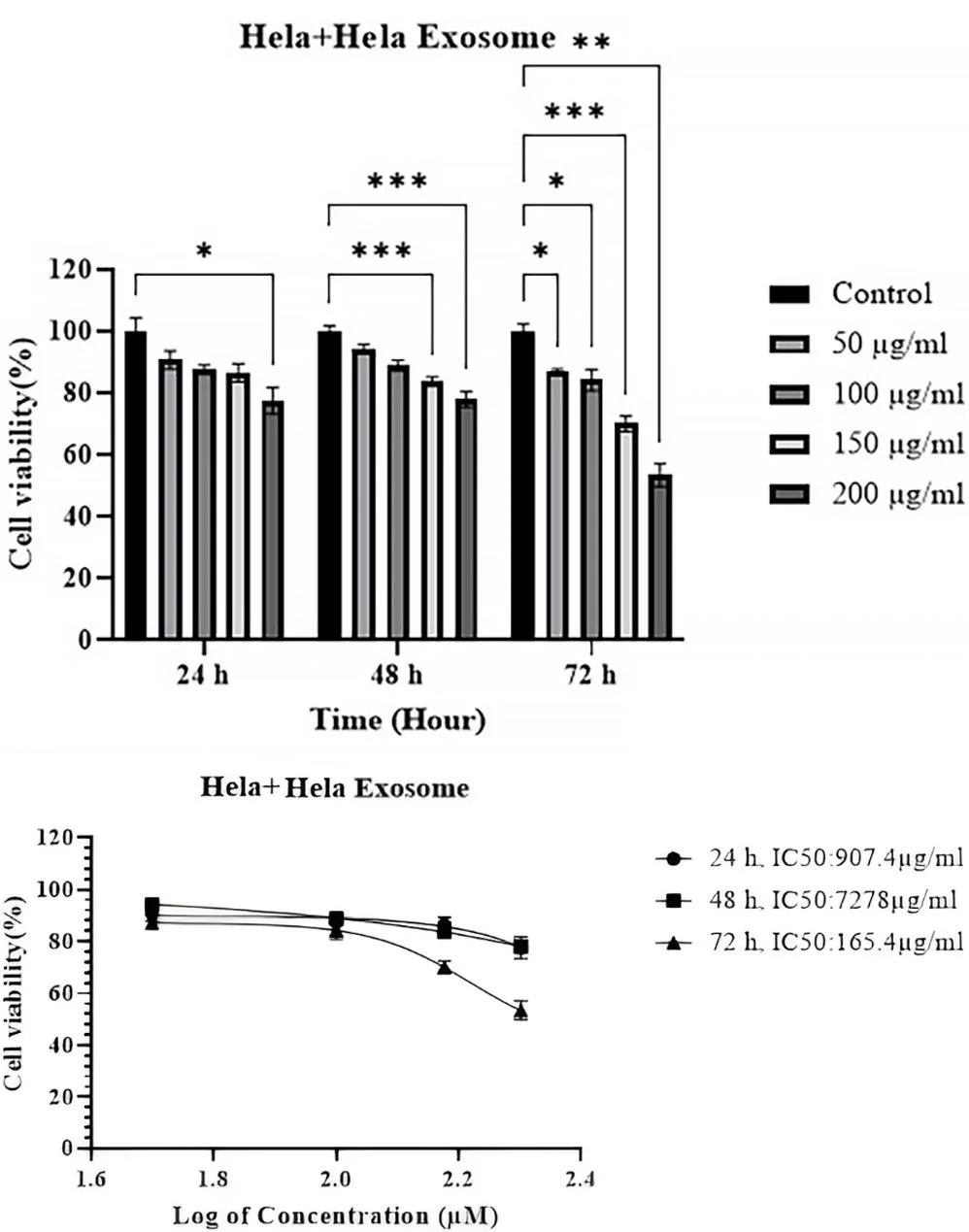
FExosome-mediated cytotoxicity in HeLa cells. (a) Cell viability assessed by MTT assay following treatment with increasing concentrations of exosomes (50–200 µg/mL) for 24, 48, and 72 h. Data are presented as mean ± SD (n = 3). We performed statistical analysis using one-way ANOVA followed by Tukey’s post hoc test (*P < 0.05, **P < 0.01, ***P < 0.001, ****P < 0.0001). (b) Dose-response curve demonstrating dose- and time-dependent reductions in cell viability, with calculated IC50 values of 907.4 µg/mL (24 h), 727.8 µg/mL (48 h), and 165.4 µg/mL (72 h).

 The dose-response curve fitting identified the exosome half-maximal inhibitory concentration (IC50) at 907.4 µg/mL at 24 hours, 727.8 µg/mL at 48 hours, and a decrease to 165.4 µg/mL at 72 hours (Figure 2b). Of note, the values observed among treatment groups and time remain significant, indicating a dose/time-dependent relationship. Based on these results, 166 µg/mL at 72 hours was selected as an ideal set-up for the present study, with enough cytotoxic activity balanced against decent ratios of cell survivability.

 A clear dose- and time-dependent decline in cell viability was observed across all tested concentrations. The gradual reduction in viability with higher doses of cisplatin (or exosomes) reflects a consistent dose–response pattern, reinforcing the suitability and reliability of the chosen concentration range for determining the IC50 value.

###  Characterization of HeLa-Derived Exosomes

 To confirm the identity and purity of the isolated exosomes, we performed comprehensive characterization using Dynamic Light Scattering (DLS), Transmission Electron Microscopy (TEM), Scanning Electron Microscopy (SEM), and Western blot analysis as previously described.

####  Dynamic Light Scattering (DLS)

 The size distribution and concentration of exosomes were assessed using DLS (Malvern Zetasizer, UK). Isolated exosomes exhibited a peak size range of 40–210 nm, consistent with the expected size of extracellular vesicles (Figure 3a). The polydispersity index (PDI) was < 0.2, indicating a homogeneous population.

**Figure 3:**
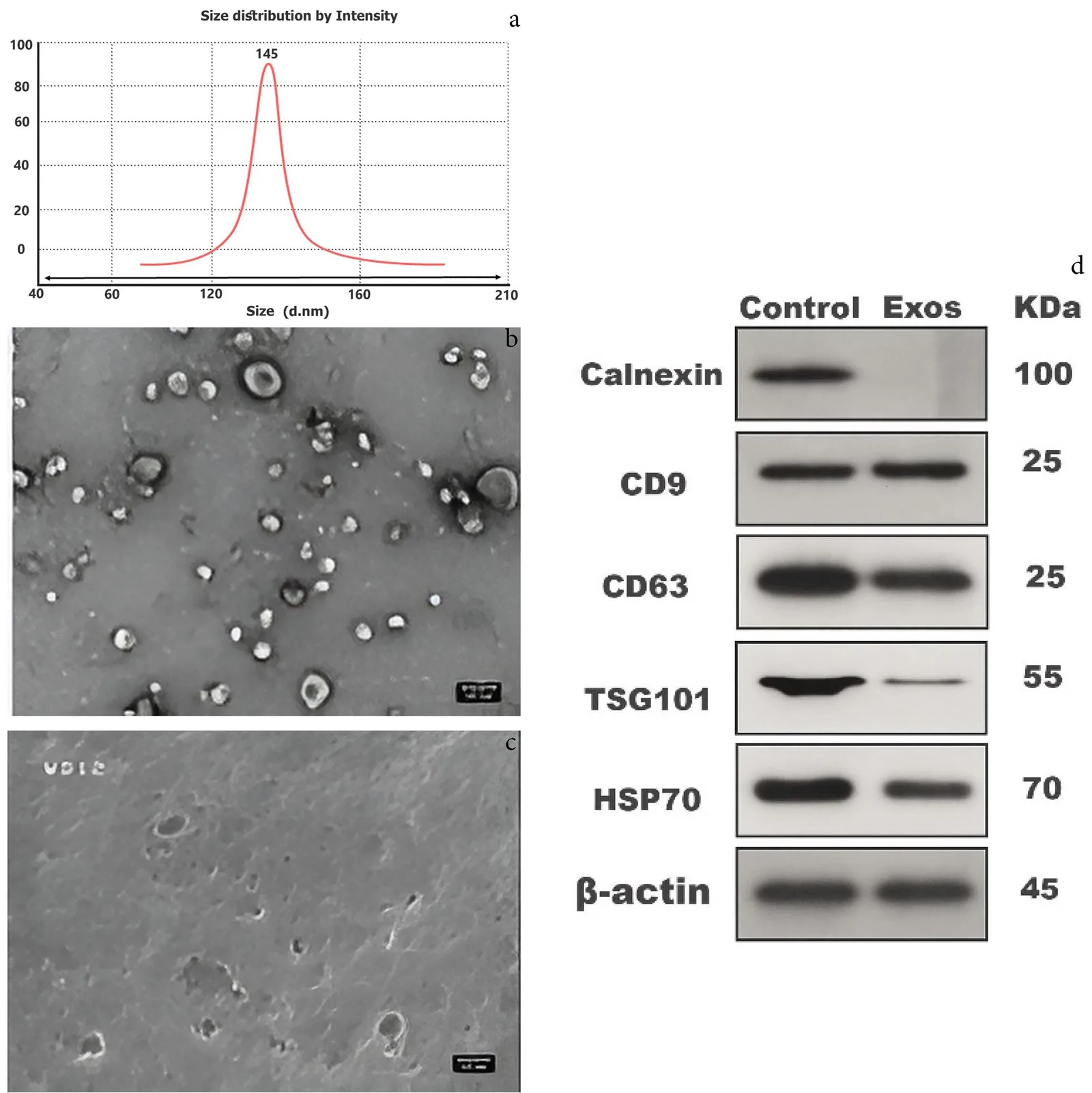
Characterization of isolated exosomes. (a) Size distribution profile by DLS, demonstrating a peak diameter of ~145 nm. (b) TEM image showing spherical, membrane-bound vesicles (scale bar: 100 nm); arrows indicate intact membranes. (c) SEM analysis confirming the characteristic cup-shaped morphology (scale bar: 100 nm). (d) Western blot analysis of exosomal markers (CD63, CD9, HSP70, and TSG101) alongside the negative control for cellular contamination (Calnexin).

####  Transmission Electron Microscopy (TEM)

 TEM (Hitachi H9500, Japan) was performed to visualize exosome morphology and membrane integrity. Samples were negatively stained with 2% uranyl acetate and imaged at 80 kV. Exosomes displayed a spherical, cup-shaped morphology with intact lipid bilayers (Figure 3b).

####  Scanning Electron Microscopy (SEM)

 SEM (ZEISS, EVO, Germany) further confirmed exosome surface morphology and size distribution. Samples were fixed with 2.5% glutaraldehyde, dehydrated in an ethanol gradient, and sputter-coated with gold before imaging. Exosomes appeared as round, membrane-bound vesicles with diameters consistent with DLS data (Figure 3c).

####  Western Blot Analysis

 Exosomal markers were verified by Western blot using anti-CD63, anti-CD9, HSP70, anti-TSG101, and calnexin antibodies (Abcam, USA). The lack of Calnexin expression (a negative exosomal marker) confirmed the absence of cellular contamination supporting the purity of the isolated exosomes. Calnexin (a negative marker for exosomes) was undetectable, confirming exosome purity (Figure 3d).

###  Gene Expression Analysis by Real-Time PCR

 Utilizing quantitative real-time PCR, we assessed the relative mRNA expression of several apoptosis, immune, metastatic, and stress-related genes to study the impacts of [Cisplatin vs Exosome exposure]. Expression levels were corrected to the housekeeping gene B2M, and the 2^−ΔCt^ technique was used to estimate fold variations.

 While the anti-apoptotic gene BCL2 showed a small but non-significant downregulation, the pro-apoptotic gene BAX was dramatically upregulated (**P < 0.01). Supporting the activation of apoptosis, expression of Caspase3—a major effector in the apoptotic pathway—also showed a significant increase (*P < 0.05).

 BIRC5 (Survivin) showed a significant downregulation (*P* < 0.05) among the genes linked with immune modulation and cell survival, PD-L1 expression did not show a statistically significant change in the initial overall analysis. However, subsequent group-wise comparisons revealed a consistent downregulation pattern, particularly in response to exosome and combination treatments.

 In addition, TNF-α, a key pro-inflammatory cytokine, was significantly elevated (**P < 0.01), which points to a possible inflammatory response started by the treatment. P53, a central cell cycle and apoptosis regulator, was upregulated (*P < 0.05), indicating involvement in stress-induced signaling. In contrast, HSP90, a molecular chaperone, revealed minimal changes in expression, implying a limited heat shock response under the experimental conditions.

 The gene expression profile indicates a pro-apoptotic and pro-inflammatory shift in response to treated exosomes, with concurrent modulation of survival and immune-related pathways.

 To facilitate a clearer interpretation of the experimental outcomes, Figures 4–10 include several panels illustrating different aspects of the analyses performed in this study. Among these, panel “c” presents the quantitative results that summarize the principal findings of the experiments. Therefore, the main comparisons within each cell line (HeLa and HEK293) as well as the comparisons between these two cell lines were primarily based on the data shown in panel “c”. The other panels are provided to visually support the experimental observations and to complement the quantitative analyses. Accordingly, in the Results and Discussion sections, emphasis has been placed on the data presented in panels “c” of Figures 4–10, and the corresponding findings are interpreted and discussed in an integrated manner to provide a coherent understanding of the observed cellular responses.

**Figure 4:**
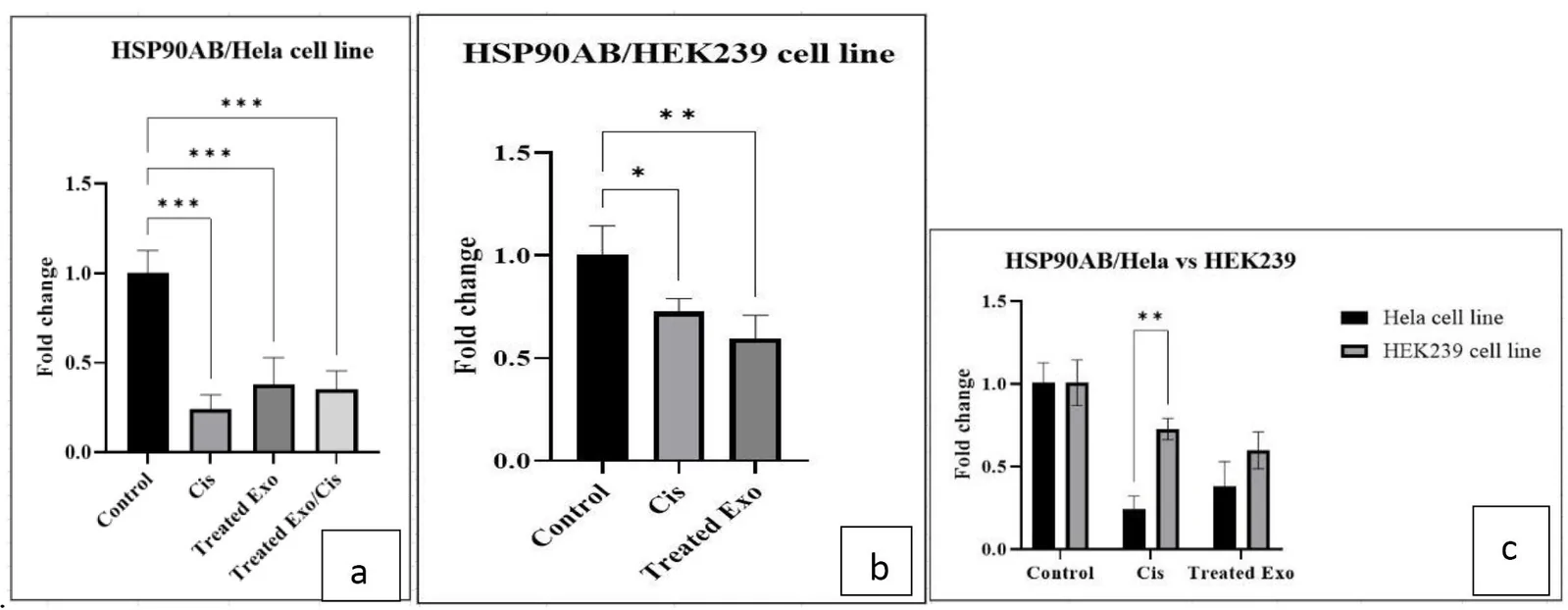
**a.** Expression level of HSP90AB in HeLa cells following different treatments. Relative fold change of HSP90AB gene expression was evaluated in HeLa cells after treatment with cisplatin (Cis), exosomes derived from treated cells (Treated Exo), and a combination of both (Treated Exo/Cis). Results revealed significant downregulation of HSP90AB expression in all treated groups compared to the control. Data are presented as mean ± SD (n = 3). Statistical analysis was performed using one-way ANOVA followed by Tukey’s post hoc test. Significance levels are indicated as follows: *P < 0.05, **P < 0.01, ***P < 0.001, ****P < 0.0001. **b.** Expression level of HSP90AB in HEK293 cells under different treatment conditions. Quantitative analysis of HSP90AB gene expression in HEK293 cells treated with cisplatin (Cis) and exosomes from treated cells (Treated Exo). Both treatment groups revealed a significant decrease in HSP90AB expression compared to the control. Data are presented as mean ± SD (n = 3). Statistical analysis was performed using one-way ANOVA followed by Tukey’s post hoc test. Significance levels are indicated as follows: *P < 0.05, **P < 0.01, ***P < 0.001, ****P < 0.0001. **c.** Comparative analysis of HSP90AB expression between HeLa and HEK293 cells. Fold change in HSP90AB expression was compared between HeLa and HEK293 cells under control, cisplatin-treated (Cis), and exosome-treated (Treated Exo) conditions. A statistically significant difference in gene expression was observed between cell lines in the Cis-treated group. Data are presented as mean ± SD (n = 3). Statistical analysis was performed using one-way ANOVA followed by Tukey’s post hoc test. Significance levels are indicated as follows: *P < 0.05, **P < 0.01, ***P < 0.001, ****P < 0.0001

**Figure 5:**
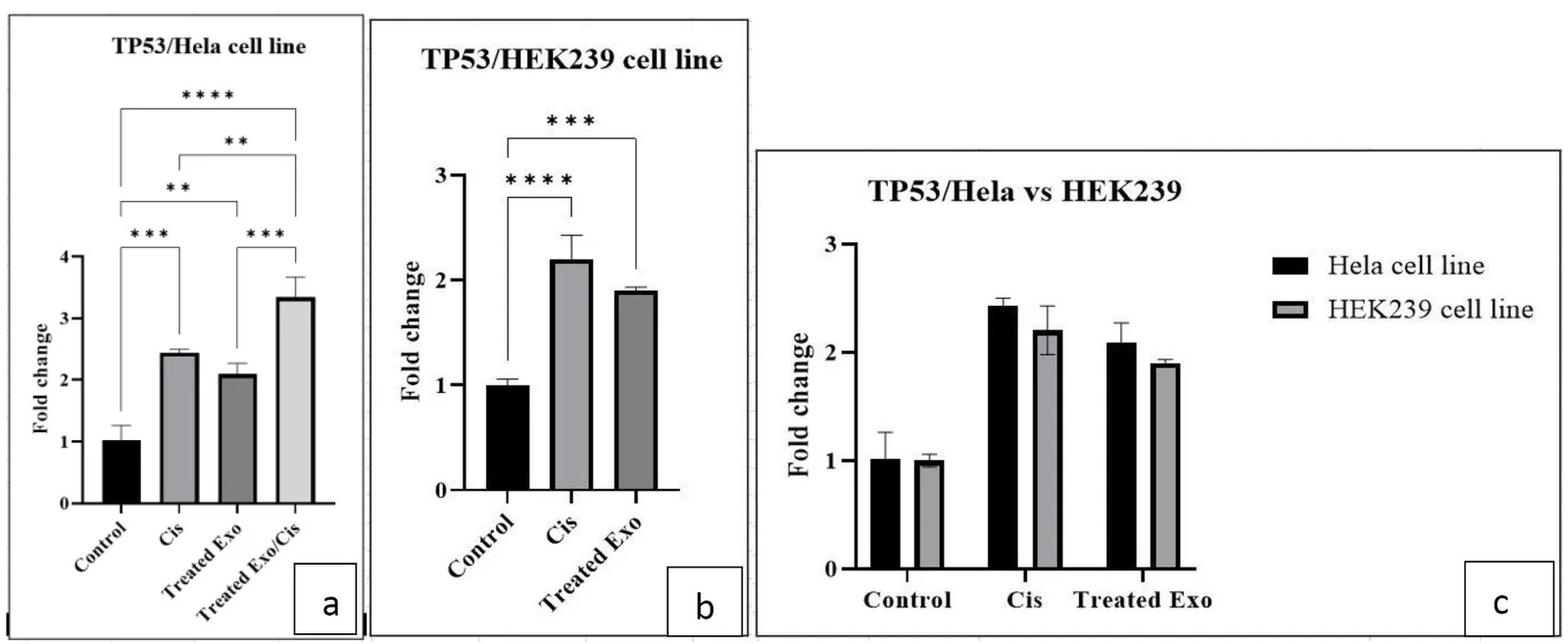
**a.** TP53 gene expression in HeLa cells following treatment with cisplatin and/or exosomes. Quantitative real-time PCR analysis of TP53 mRNA levels in HeLa cells treated with cisplatin (Cis), exosomes (Treated Exo), or a combination of both (Treated Exo/Cis). Expression levels are shown as fold change relative to the untreated control. Data are presented as mean ± SD (n = 3). Statistical analysis was performed using one-way ANOVA followed by Tukey’s post hoc test. Significance levels are indicated as follows: *P < 0.05, **P < 0.01, ***P < 0.001, ****P < 0.0001. **b. **TP53 gene expression in HEK293 cells following treatment with cisplatin and exosomes. qRT-PCR analysis of TP53 mRNA expression in HEK293 cells after treatment with cisplatin (Cis) or exosomes (Treated Exo). Expression is normalized to the untreated control group. Data are presented as mean ± SD (n = 3). Statistical analysis was performed using one-way ANOVA followed by Tukey’s post hoc test. Significance levels are indicated as follows: *P < 0.05, **P < 0.01, ***P < 0.001, ****P < 0.0001. **c.** Comparison of TP53 gene expression between HeLa and HEK293 cell lines. Fold change in TP53 expression following cisplatin and exosome treatment in HeLa and HEK293 cells. qRT-PCR results demonstrate differential TP53 induction between the cancerous (HeLa) and non-cancerous (HEK293) cell lines. Data are presented as mean ± SD (n = 3). Statistical analysis was performed using one-way ANOVA followed by Tukey’s post hoc test. Significance levels are indicated as follows: *P < 0.05, **P < 0.01, ***P < 0.001, ****P < 0.0001

**Figure 6:**
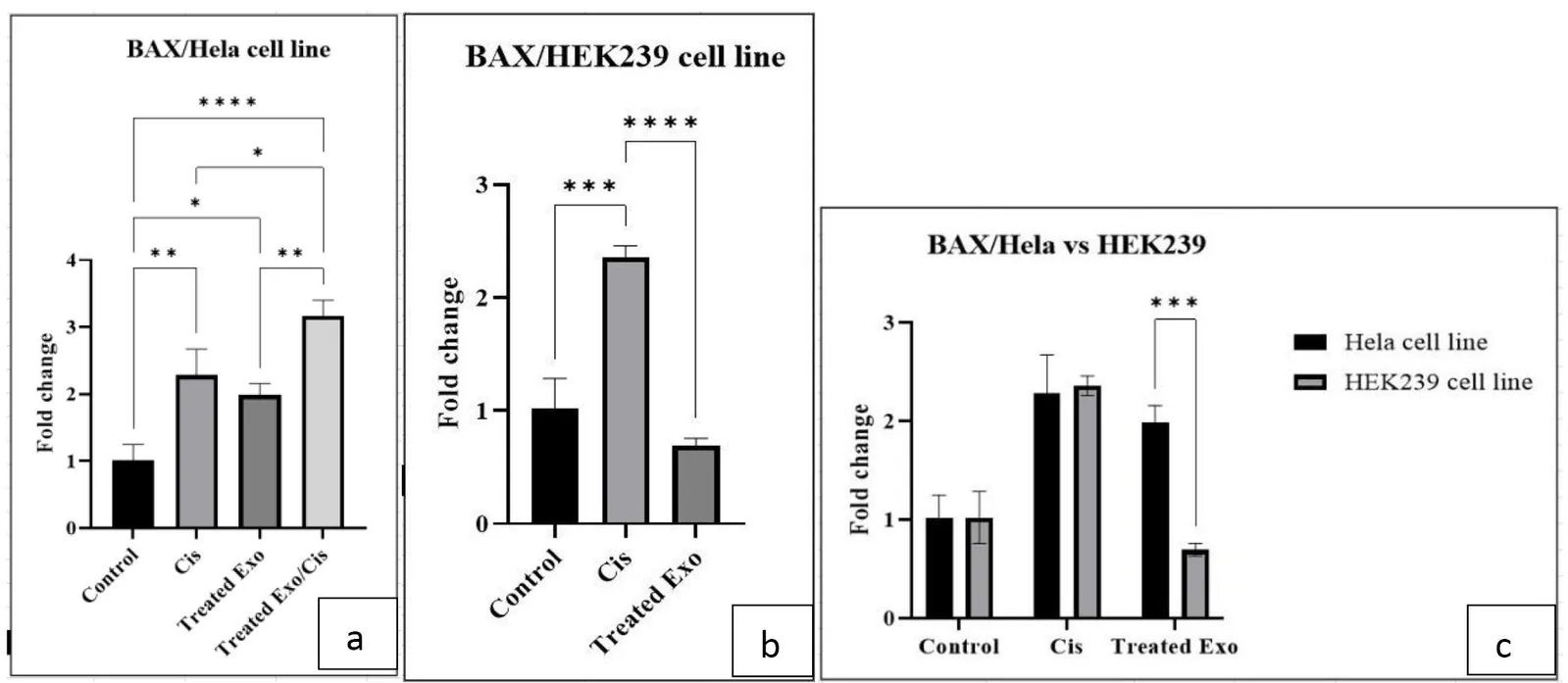
**a**. Relative expression of the BAX gene in HeLa cells under different treatment conditions. HeLa cells were treated with cisplatin (Cis), treated exosomes (Treated Exo), or a combination of both (Treated Exo + Cis). The expression level of BAX was measured by qRT-PCR and normalized to the control group. Data are presented as mean ± SD (n = 3). Statistical analysis was performed using one-way ANOVA followed by Tukey’s post hoc test. Significance levels are indicated as follows: *P < 0.05, **P < 0.01, ***P < 0.001, ****P < 0.0001. **b**. BAX gene expression in HEK293 cells following treatment with cisplatin or treated exosomes. HEK293 cells were exposed to cisplatin (Cis) or treated with exosomes (Treated Exo). qRT-PCR analysis was used to evaluate BAX mRNA levels relative to those of the control group. Data are presented as mean ± SD (n = 3). Statistical analysis was performed using one-way ANOVA followed by Tukey’s post hoc test. Significance levels are indicated as follows: *P < 0.05, **P < 0.01, ***P < 0.001, ****P < 0.0001. **c**. Comparison of BAX gene expression between HeLa and HEK293 cell lines under different conditions. Both HeLa and HEK293 cells were treated with cisplatin (Cis) or treated with exosomes (Treated Exo). BAX expression levels were quantified using qRT-PCR. The comparison indicates that treated exosomes substantially induced BAX expression in HeLa cells but not HEK293 cells. Data are presented as mean ± SD (n = 3). Statistical analysis was performed using one-way ANOVA followed by Tukey’s post hoc test. Significance levels are indicated as follows: *P < 0.05, **P < 0.01, ***P < 0.001, ****P < 0.0001

**Figure 7:**
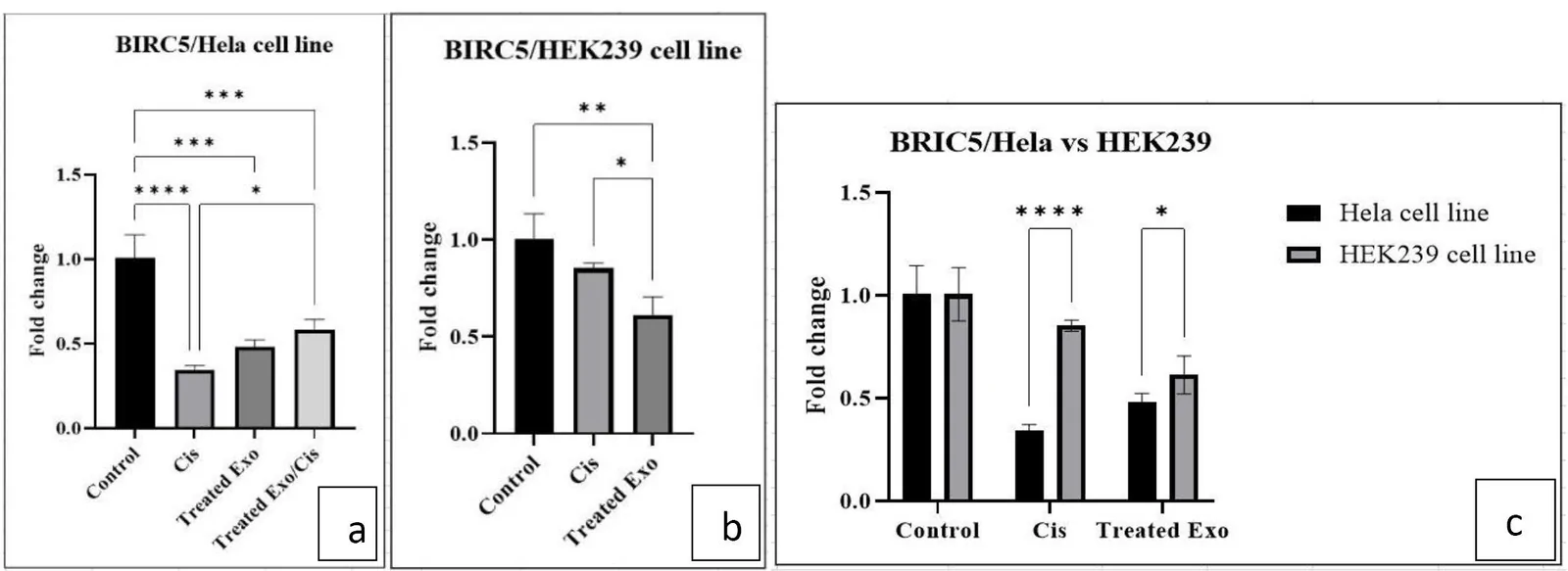
**a.** Relative expression of BIRC5 in the HeLa cell line under different treatment conditions. HeLa cells were treated with cisplatin (Cis), exosomes derived from treated cells (Treated Exo), and a combination of both (Treated Exo/Cis). The expression level of BIRC5 was assessed by qRT-PCR and normalized to the control group (untreated cells). Data are presented as mean ± SD (n = 3). Statistical analysis was performed using one-way ANOVA followed by Tukey’s post hoc test. Significance levels are indicated as follows: *P < 0.05, **P < 0.01, ***P < 0.001, ****P < 0.0001. **b.** Relative expression of BIRC5 in HEK293 cell line following cisplatin and exosome treatments. HEK293 cells were exposed to cisplatin (Cis) or treated with exosomes (Treated Exo), and BIRC5 gene expression was analyzed using qRT-PCR. Expression levels were normalized to the control (untreated) group. Data are presented as mean ± SD (n = 3). Statistical analysis was performed using one-way ANOVA followed by Tukey’s post hoc test. Significance levels are indicated as follows: *P < 0.05, **P < 0.01, ***P < 0.001, ****P < 0.0001. **c.** Comparison of BIRC5 expression between HeLa and HEK293 cell lines. Expression of BIRC5 was compared between HeLa and HEK293 cells under three conditions: untreated (Control), cisplatin-treated (Cis), and exosome-treated (Treated Exo). The results demonstrate a substantially stronger downregulation in HeLa cells compared to HEK293 cells, especially under Cis treatment. Data are presented as mean ± SD (n = 3). Statistical analysis was performed using one-way ANOVA followed by Tukey’s post hoc test. Significance levels are indicated as follows: *P < 0.05, **P < 0.01, ***P < 0.001, ****P < 0.0001

**Figure 8:**
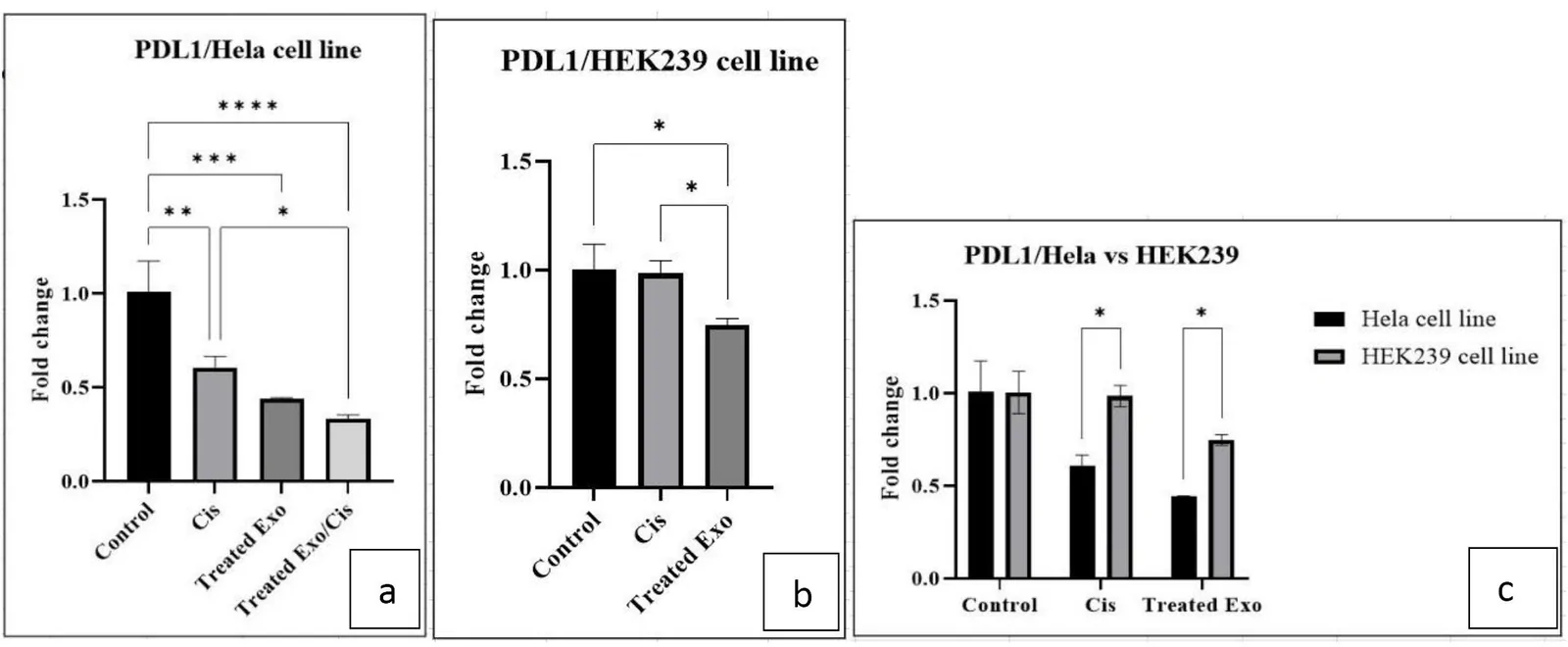
**a**. PD-L1 expression levels in HeLa cells under different treatments. Bar graph demonstrating fold change in PD-L1 expression in HeLa cells after treatment with Cisplatin (Cis), exosomes derived from treated cells (Treated Exo), and a combination of both (Treated Exo/Cis). All treatments substantially reduced PD-L1 levels compared to control cells, with the combination treatment demonstrating the greatest effect (****P < 0.0001, ***P < 0.001, **P < 0.01, *P < 0.05). Data are presented as mean ± SD (n = 3). Statistical analysis was performed using one-way ANOVA followed by Tukey’s post hoc test. **b.** PD-L1 expression levels in HEK293 cells after treatment. Fold change in PD-L1 expression in HEK293 cells treated with Cisplatin (Cis) and exosomes from treated cells (Treated Exo). Both treatments resulted in a significant decrease in PD-L1 levels compared to the control group (*P < 0.05). Data are presented as mean ± SD (n = 3). Statistical analysis was performed using one-way ANOVA followed by Tukey’s post hoc test. Significance levels are indicated as follows: *P < 0.05, **P < 0.01, ***P < 0.001, ****P < 0.0001. **c**. Comparison of PD-L1 expression between HeLa and HEK293 cell lines. Bar chart comparing fold changes in PD-L1 expression in HeLa and HEK293 cells under control, Cisplatin, and exosome treatment conditions. PD-L1 downregulation was substantially more pronounced in HeLa cells compared to HEK293 cells in response to both Cis and Treated Exo (*P < 0.05). Data indicate differential sensitivity of cell lines to PD-L1 modulation. Data are presented as mean ± SD (n = 3). Statistical analysis was performed using one-way ANOVA followed by Tukey’s post hoc test. Significance levels are indicated as follows: *P < 0.05, **P < 0.01, ***P < 0.001, ****P < 0.0001

**Figure 9:**
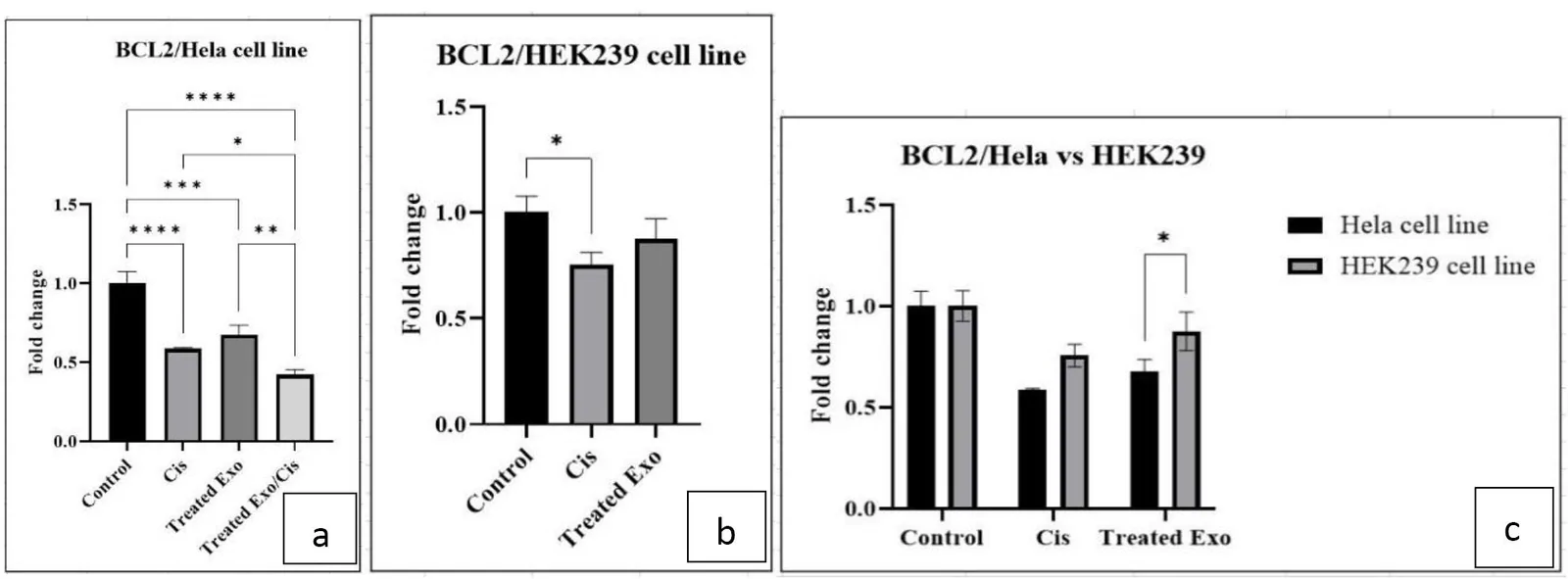
**a**. Relative expression of BCL2 in HeLa cells under different treatment conditions. HeLa cells were treated with cisplatin (Cis), exosomes (Treated Exo), or a combination of both (Treated Exo/Cis). BCL2 expression levels were measured and presented as fold change relative to the untreated control group. Significant reductions in BCL2 expression were observed in all treated groups compared to control, with the combined Exo/Cis treatment demonstrating the most pronounced downregulation. Data are presented as mean ± SD (n = 3). Statistical analysis was performed using one-way ANOVA followed by Tukey’s post hoc test. Significance levels are indicated as follows: *P < 0.05, **P < 0.01, ***P < 0.001, ****P < 0.0001. **b**. BCL2 expression levels in HEK293 cells following treatment. HEK293 cells were exposed to cisplatin (Cis) or exosomes (Treated Exo), and BCL2 expression was assessed relative to the control. A slight but significant decrease in BCL2 expression was observed only in the Cis-treated group. No significant change was detected in the exosome-treated group. Data are presented as mean ± SD (n = 3). Statistical analysis was performed using one-way ANOVA followed by Tukey’s post hoc test. Significance levels are indicated as follows: *P < 0.05, **P < 0.01, ***P < 0.001, ****P < 0.0001. **c**. Comparison of BCL2 expression between HeLa and HEK293 cell lines. The relative expression of BCL2 was compared between HeLa (black bars) and HEK293 (gray bars) cell lines under control, Cis, and Treated Exo conditions. A significant difference was observed in the Treated Exo group, where HEK293 cells revealed higher BCL2 expression than HeLa cells. Data are presented as mean ± SD (n = 3). Statistical analysis was performed using one-way ANOVA followed by Tukey’s post hoc test. Significance levels are indicated as follows: *P < 0.05, **P < 0.01, ***P < 0.001, ****P < 0.0001

**Figure 10:**
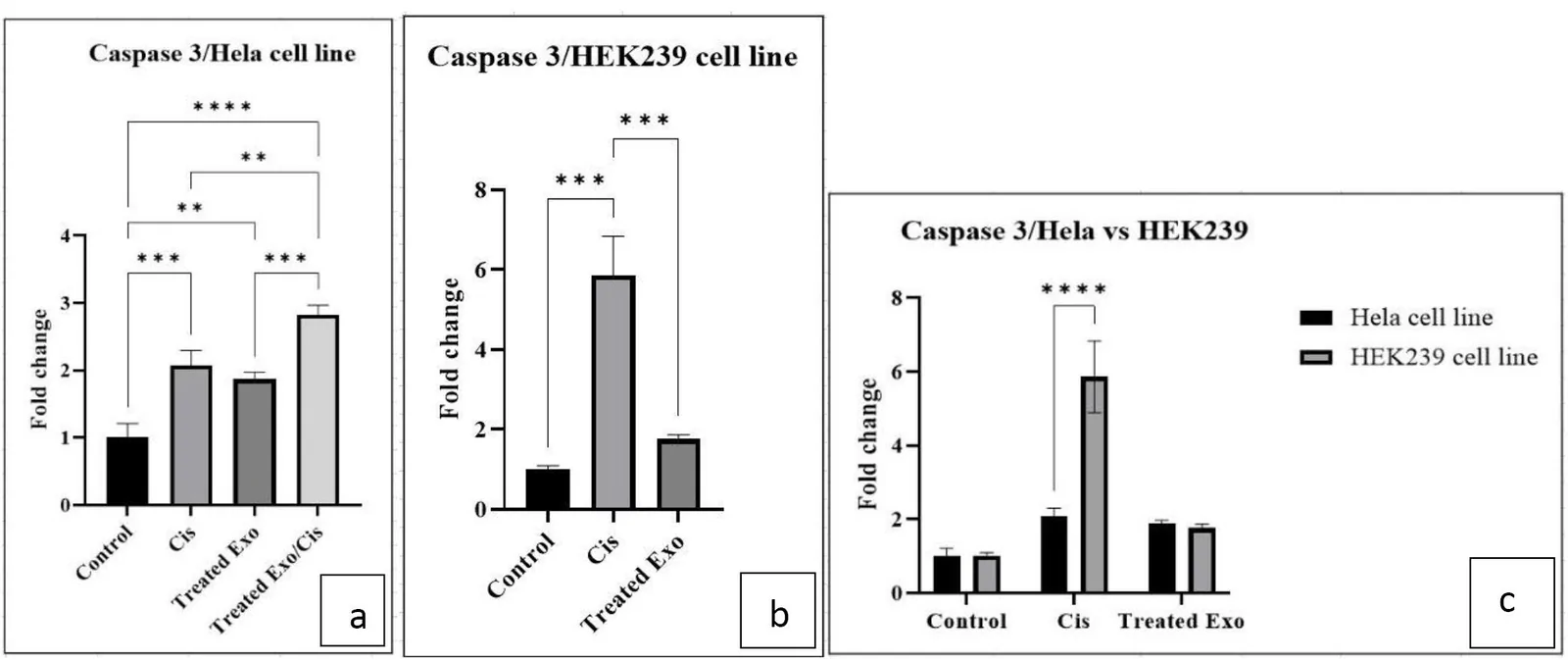
**a**. Caspase-3 expression in the HeLa cell line after different treatments. The fold change in caspase-3 mRNA expression was assessed in HeLa cells treated with Cisplatin (Cis), Exosome (Treated Exo), and a combination of both (Treated Exo/Cis) compared to the untreated control group. Data are presented as mean ± SD (n = 3). Statistical analysis was performed using one-way ANOVA followed by Tukey’s post hoc test. Significance levels are indicated as follows: *P < 0.05, **P < 0.01, ***P < 0.001, ****P < 0.0001. **b**. Caspase-3 expression in the HEK293 cell line after different treatments. The expression level of caspase-3 was measured in HEK293 cells following treatment with Cisplatin (Cis) and Exosome (Treated Exo) in comparison to the control. Cisplatin markedly induced caspase-3 expression. Data are presented as mean ± SD (n = 3). Statistical analysis was performed using one-way ANOVA followed by Tukey’s post hoc test. Significance levels are indicated as follows: *P < 0.05, **P < 0.01, ***P < 0.001, ****P < 0.0001. **c.** Comparison of caspase-3 expression between HeLa and HEK293 cell lines. Fold change in caspase-3 expression was compared between HeLa and HEK293 cells under Control, Cisplatin (Cis), and Exosome (Treated Exo) treatments. HEK293 cells revealed substantially higher caspase-3 expression upon cisplatin treatment compared to HeLa cells. Data are presented as mean ± SD (n = 3). Statistical analysis was performed using one-way ANOVA followed by Tukey’s post hoc test. Significance levels are indicated as follows: *P < 0.05, **P < 0.01, ***P < 0.001, ****P < 0.0001

####  Differential Expression of HSP90AB in HeLa Cells

 As shown in Figure 4a, HSP90AB expression in HeLa cells was significantly downregulated following the applied treatments. Compared with the control group, cisplatin treatment resulted in a marked and statistically significant decrease in HSP90AB expression. Treatment with exosomes alone also reduced the expression level of this gene, although the magnitude of reduction was lower than that observed with cisplatin treatment. Similarly, the combined Exo/Cis treatment maintained HSP90AB expression at a level significantly below the control group. These findings indicate that the applied treatments suppress HSP90AB expression in HeLa cells, suggesting a potential alteration in cellular stress-response pathways in this cancer cell line.

####  HSP90AB Response in HEK293 Cells

 In HEK293 cells (Figure 4b), a comparable but less pronounced pattern was observed. Both cisplatin and exosome treatments led to a reduction in HSP90AB expression compared with the control group. Statistical analysis indicated that these decreases were significant relative to the control condition. However, the magnitude of downregulation was smaller than that observed in HeLa cells, indicating that HEK293 cells exhibited a milder transcriptional response to the treatments.

####  Comparative Analysis Between HeLa and HEK293 Cell Lines

 The comparative analysis between the two cell lines (Figure 4c) further confirmed the differential response of HSP90AB expression to the treatments. Although both cell lines demonstrated reduced expression levels relative to their respective controls, the decrease was more pronounced in HeLa cells than in HEK293 cells, particularly under cisplatin treatment. This observation suggests that the cancerous HeLa cells may be more susceptible to treatment-induced modulation of HSP90AB expression compared with the non-cancerous HEK293 cells, highlighting the potential influence of cellular context on the regulation of stress-related genes.

 TP53 Gene Expression is Upregulated Following Treatment with Cisplatin and Exosomes in HeLa and HEK293 Cell Lines.

 Quantitative real-time PCR (qRT-PCR) on HeLa and HEK293 cell lines was done to assess the influence of several therapies on TP53 gene expression.

 To guarantee reproducibility and statistical dependability, every experiment was repeated three times.

 Compared to the untreated control, TP53 expression in the HeLa cell line (Figure 5a) was markedly increased in response to all treatment groups. Cells treated with cisplatin (Cis) showed a notable increase (approximately 2.5-fold; ***P < 0.001), whereas those treated with exosomes alone (Treated Exo) also showed significant upregulation. (~2.2-fold; **P < 0.01). The combination of cisplatin and exosome therapy (Treated Exo/Cis) resulted in the highest expression level (around 3.5-fold increase; ****P < 0.0001), much surpassing every one of the separate therapies (***P < 0.001 vs. Cis and Treated Exo). This implies a synergistic effect of the combined treatment.

 In the HEK293 cell line (Figure 5b), cisplatin as well as exosome treatments resulted in a notable increase in TP53 expression relative to the control. Exosome-treated cells showed a ~2-fold rise (***P < 0.001), whereas Cis therapy produced a ~2.3-fold increase (****P < 0.0001). The magnitude of upregulation was somewhat less, even if the pattern resembled that seen in HeLa cells.

 Comparatively analyzing the two cell lines (Figure 5c), HeLa cells always displayed greater TP53 expression in response to both Cis and Exo conditions, in comparison to HEK293 cells. This variation may be indicative of different biological reactions between non-cancerous (HEK293) and cancerous (HeLa) cell settings.

 The results suggest that, particularly in combination, cisplatin and exosome treatments successfully increase TP53 expression, with a greater impact seen in the HeLa cancer cell model.

 No statistical comparisons between cell lines are indicated in this figure; error bars represent standard deviation.

####  The Expression Levels of the Pro-Apoptotic Gene BAX were Evaluated in HeLa and HEK293 Cell Lines Under Different Treatment Conditions Using qRT-PCR, and the Results are Presented as Fold Change Relative to the Control Group.

 All in HeLa cells (Figure 6a), cisplatin (Cis), treated exosomes, and a combination of treated exosomes and cisplatin (Treated Exo + Cis) caused notable upregulation of BAX expression, which was markedly different (**P < 0.01 to ****P < 0.0001) from the control group. Suggesting a synergistic pro-apoptotic impact of the combination, the Treated Exo + Cis group showed the greatest expression—more than a 3.5-fold increase (****P < 0.0001)— (Figure 6a).

 On the other hand, in HEK293 cells (Figure 6b), cisplatin treatment by itself showed marked upregulation of BAX expression in relation to control (***P < 0.001), whereas treated exosomes alone showed significant Downregulation of BAX expression (****P < 0.0001), showing that non-cancerous cells may benefit from a protective or anti-apoptotic influence from exosomes (Figure 6b).

 A comparison of the two cell types revealed that treated exosomes (***P < 0.001) elicited a significantly higher BAX expression in HeLa cells than HEK293 cells. No noticeable variations were seen in either control or cisplatin settings (Figure 6c), showing that treated exosomes showed pro-apoptotic activity just on malignant cells.

####  To Evaluate the Expression Level of the BIRC5 Gene Under Different Treatment Conditions, Quantitative Analysis was Performed on HeLa and HEK293 Cell Lines.

#####  Expression of BIRC5 in HeLa Cells

 Cisplatin (Cis) treatment resulted in a decrease in the expression of BIRC5 in HeLa cells, as demonstrated in Figure 7a, compared to the control (**P < 0.01). The treated exosomes (Treated Exo) resulted in a similarly large decrease in BIRC5 expression (***P < 0.001), but the decrease was slightly less than in the Cis-only treatment. With the combination group (Treated Exo/Cis), BIRC5 expression was partially restored compared to the Cis group, but still significantly lower than control BIRC5 levels (*P < 0.05).

#####  Expression of BIRC5 in HEK293 Cells

 In Figure 7b, the HEK293 cells showed a more moderate response to the treatments. Cisplatin led to a significant decrease in BIRC5 expression compared to the control (**P < 0.01), and Treated Exo also reduced BIRC5 expression substantially (*P < 0.05). However, the overall level of gene downregulation in HEK293 cells was less pronounced than in HeLa cells.

#####  Comparative Expression Between Cell Lines

 A comparison of HeLa and HEK293 cells, as shown in Figure 7c, showed that cisplatin-induced downregulation of BIRC5 was much greater in HeLa cells than ****P < 0.0001 in HEK293 cells. Similarly, HeLa cells treated with exosomes showed considerably less BIRC5 expression than HEK293 counterparts (*P < 0.05). Between the untreated control groups of the two cell lines, no major variation was noticed.

####  To Investigate the Effect of Different Treatments on PD-L1 Expression, We Analyzed Fold Changes in PD-L1 Levels Across HeLa and HEK293 Cell Lines Under Various Experimental Conditions.

#####  PD-L1 Expression in HeLa Cells

 Following the initial overall gene expression screening, a more detailed group-wise analysis was performed to clarify PD-L1 regulatory patterns under specific treatment conditions. PD-L1 expression dramatically dropped in all treated groups compared to the control group, as seen in Figure 8A. While exosome-treated cells showed a significantly larger decrease (***P < 0.001), treatment with cisplatin only resulted in a substantial decrease (**P < 0.01). The combined Treatment of Cisplatin (Exo/Cis) and exosomes resulted in the most significant drop in PD-L1 expression compared to the control (****P < 0.0001), showing a possible synergistic effect.

 PD-L1 expression in HEK293 cells:

 In the HEK293 cell line (Figure 8B), PD-L1 levels also decreased in response to treatment. While Cisplatin alone induced a slight reduction (*P < 0.05), exosome treatment caused a more evident suppression of PD-L1 (*P < 0.05), indicating that exosomes have a regulatory role even in non-cancerous or less aggressive cell lines.

 Comparison between HeLa and HEK293 cell lines:

 As seen in Figure 8C, a comparison of PD-L1 expression between the two cell lines resulted in a much larger response from the HeLa cells. Even though there was a reduction in PD-L1 expression in HeLa Cisplatin and exosome-treated motivators when compared to HEK293 under the same conditions, this reduction was still statistically significant (*P < 0.05), demonstrating greater sensitivity of the cancer cell line to the treatment.

 Combining all the above findings, Cisplatin therapy and exosome therapies are effective at downregulating PD-L1 expression, where the added values of the concurrent therapies made the most pronounced effect when examined in the HeLa cells.

####  To Evaluate the Expression of BCL2, a Key Anti-Apoptotic Gene, Under Various Treatments, We Analyzed its Fold Change in Hela And HEK293 Cell Lines.

 Compared to the control group (****P < 0.0001), treatment with cisplatin (Cis) clearly decreases BCL2 expression in HeLa cells (Figure 9a), cells shown a substantive decrease with CIS (***P < 0.001), but the combined Exo/Cis therapy showed the greatest downregulation of BCL2 (****P < 0.0001 vs. **P < 0.01 vs Exo treated, control;), again indicating a synergistic effect of cisplatin and exosomes in inhibiting anti-apoptotic signals.

 Conversely, the HEK293 cell line (Figure 9b) only mustered a slight reduction in BCL2 expression following cisplatin therapy (*P < 0.05), whereas the exosome treatment alone showed little impact and sustained a similar amount of BCL2. This description indicates less responsive non-cancerous HEK293 cells to both treatments in BCL2 downregulation.

 For comparison, between HeLa and HEK293 cell lines (Figure 9c), the treated group showed considerable differences in BCL2 expression (*P < 0.05). HEK293 cells maintained greater levels of BCL2 than HeLa cells after treatment. Compared to the control status, there are no major differences. The Control and cisplatin-treated groups both maintained constant levels of BCL2.

 Overall, these findings underscore the differential response of cancerous cells compared to non-cancerous cell lines to treatment and the potential of the combined exosomes and chemotherapeutic drugs to enhance apoptotic activity of cancer cells.

####  To Evaluate the Effect of Different Treatments on Apoptosis Induction, the Expression Level of Caspase-3 was Analyzed in HeLa and HEK293 Cell Lines.

 In the HeLa cell line (Figure 10a), treatment with cisplatin (Cis) substantially increased caspase-3 expression compared to the control group (***P < 0.001). Similarly, cells treated with exosome (Treated Exo) or a combination of exosome and cisplatin (Treated Exo/Cis) revealed elevated levels of caspase-3 expression compared to the control (**P < 0.01 and ***P < 0.001, respectively). Notably, the combination treatment (Exo/Cis) resulted in the highest fold change (~3.2-fold) among all groups, indicating a potential synergistic effect.

 In the HEK293 cell line (Figure 10b), cisplatin treatment led to a strong and significant increase in caspase-3 expression (~6-fold, ***P < 0.001), while treatment with exosomes alone resulted in a modest but significant increase (~1.8-fold, ***P < 0.001) compared to control. When comparing the two cell lines directly (Figure 10c), a substantially higher caspase-3 expression level was observed in HEK293 cells treated with cisplatin compared to HeLa cells (****P < 0.0001), indicating differential sensitivity to cisplatin-induced apoptosis. In contrast, caspase-3 expression following exosome treatment was relatively similar in both cell lines.

####  To Investigate the Effect of Treatments on TNF-ɑ Expression, Fold Change Levels Were Assessed in Both HeLa and HEK293 Cell Lines Under Various Conditions.

 Treatment with Cisplatin (Cis) significantly raised TNF-ɑ expression in the HeLa cell line (Figure 11a) compared to the control group (***P < 0.001). Exosomes originating, although to a smaller degree than Cis alone, treated cells (Treated Exo) also resulted in a statistically significant increase in TNF-alpha levels relative to the control (**P < 0.01). Coincidentally, co-treatment with exosomes and Cis (Treated Exo/Cis) showed a comparable rise to that observed with Cis alone. Statistical comparisons showed major differences between Cis and Treated Exo (*P < 0.05), as well as between Treated Exo and Treated Exo/Cis (*P < 0.05), pointing to partial modulation by exosomes.

**Figure 11:**
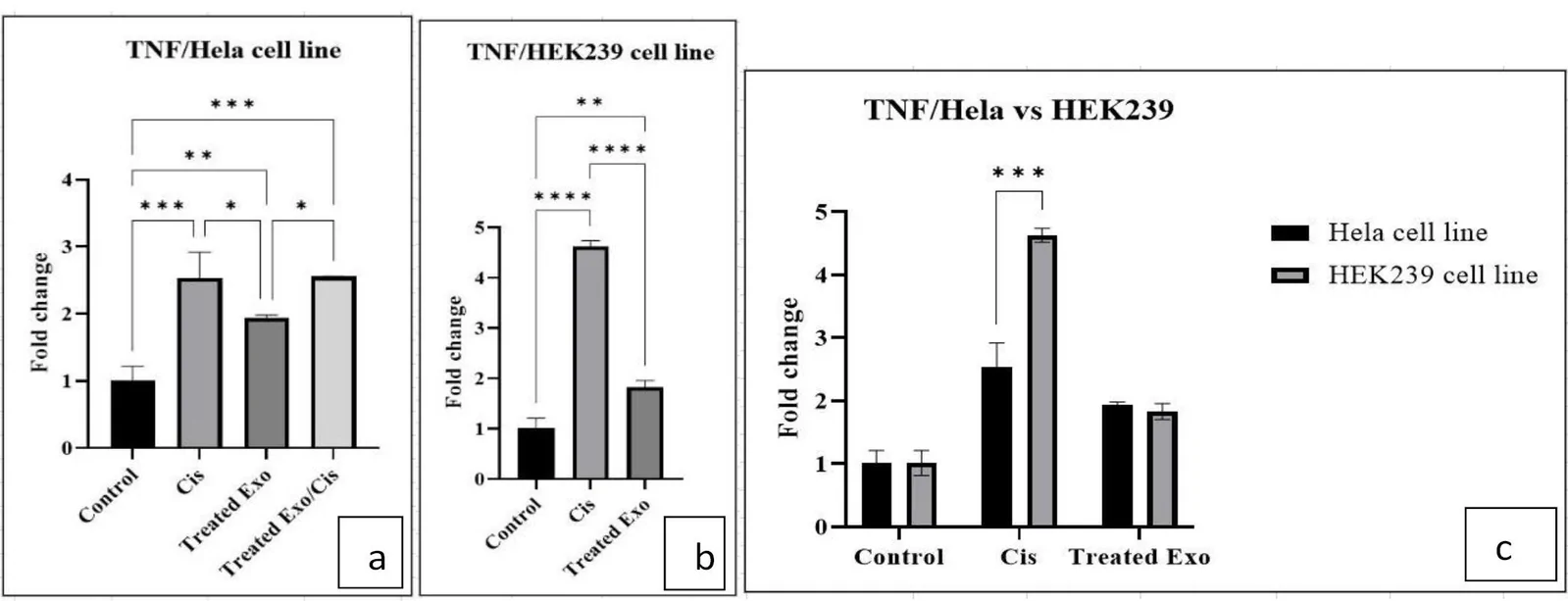
**a**. TNF-ɑ expression in HeLa cells under different treatment conditions. Bar graph representing fold change in TNF-ɑ gene expression in HeLa cells treated with Cisplatin (Cis), exosomes derived from treated cells (Treated Exo), and the combination of both (Treated Exo/Cis). TNF-ɑ expression was substantially increased in all treatment groups compared to the control. Data are presented as mean ± SD (n = 3). Statistical analysis was performed using one-way ANOVA followed by Tukey’s post hoc test. Significance levels are indicated as follows: *P < 0.05, **P < 0.01, ***P < 0.001, ****P < 0.0001. **b**. TNF-ɑ expression in HEK293 cells under different treatment conditions. Fold change in TNF-ɑ expression in HEK293 cells following treatment with Cisplatin (Cis) and Treated Exo. Cis treatment led to a strong induction of TNF-α compared to the control, while Treated Exo also substantially elevated TNF-α levels. Data are presented as mean ± SD (n = 3). Statistical analysis was performed using one-way ANOVA followed by Tukey’s post hoc test. Significance levels are indicated as follows: *P < 0.05, **P < 0.01, ***P < 0.001, ****P < 0.0001. **c**.Comparison of TNF-ɑ expression between HeLa and HEK293 cells. Comparison of TNF-ɑ fold change levels in HeLa and HEK293 cells under Control, Cisplatin (Cis), and Treated Exo conditions. Cis treatment resulted in substantially higher TNF-ɑ expression in HEK293 than in HeLa cells. Treated Exo had a similar effect in both cell lines. Data are presented as mean ± SD (n = 3). Statistical analysis was performed using one-way ANOVA followed by Tukey’s post hoc test. Significance levels are indicated as follows: *P < 0.05, **P < 0.01, ***P < 0.001, ****P < 0.0001

 Following Cis treatment in the HEK293 cell line (Figure 11b), TNF-ɑ expression was dramatically increased, almost a 5-fold rise above the control group (TNF levels in the Treated Exo group also showed a marked rise over those of the control (****P < 0.0001), albeit much less than that. Of the Cis-treated group (****P < 0.0001), showing a great but unique impact of exosomal therapy.

 Comparisons of HeLa and HEK293 cells (Figure 11c) showed that Cis caused noticeably more TNF-ɑ expression in HEK293 cells than in HeLa cells (***P < 0.001), stressing cell-type-specific sensitivity to the therapy. But under untreated exo conditions, both cell lines showed comparable degrees of TNF-α induction, suggesting that exosomal effects may be more evenly spread throughout various cell types.

 These results imply that, depending on the cell type, exosome therapy alone or in combination may modulate Cisplatin’s powerful stimulation of TNF-α expression. suggesting the possibility for targeted therapeutic strategies.

###  Effect of Cisplatin Alone and Exosomes Isolated from Cis-Treated HeLa Cells on the Apoptosis of Naïve Hela Cells. 

 Flow cytometry was used to determine the apoptotic rate of HeLa cells in response to different treatments: control exosomes (exosomes derived from naïve HeLa cell line), Cisplatin, exosomes from Cis-treated cells, and their combinations. Figure 12 shows that the % of apoptosis in HeLa cells was: 0.4% in the control groups; 60.7% in groups treated with Cis, 57.6% in groups treated with Treated Exo, and 59.3% in groups treated with Cis-Treated Exo. In comparison to the control and control-Exo groups, the apoptosis rate in HeLa cells was substantially higher in Cis, Cis-Exo, and Treated-Exo.

**Figure 12:**
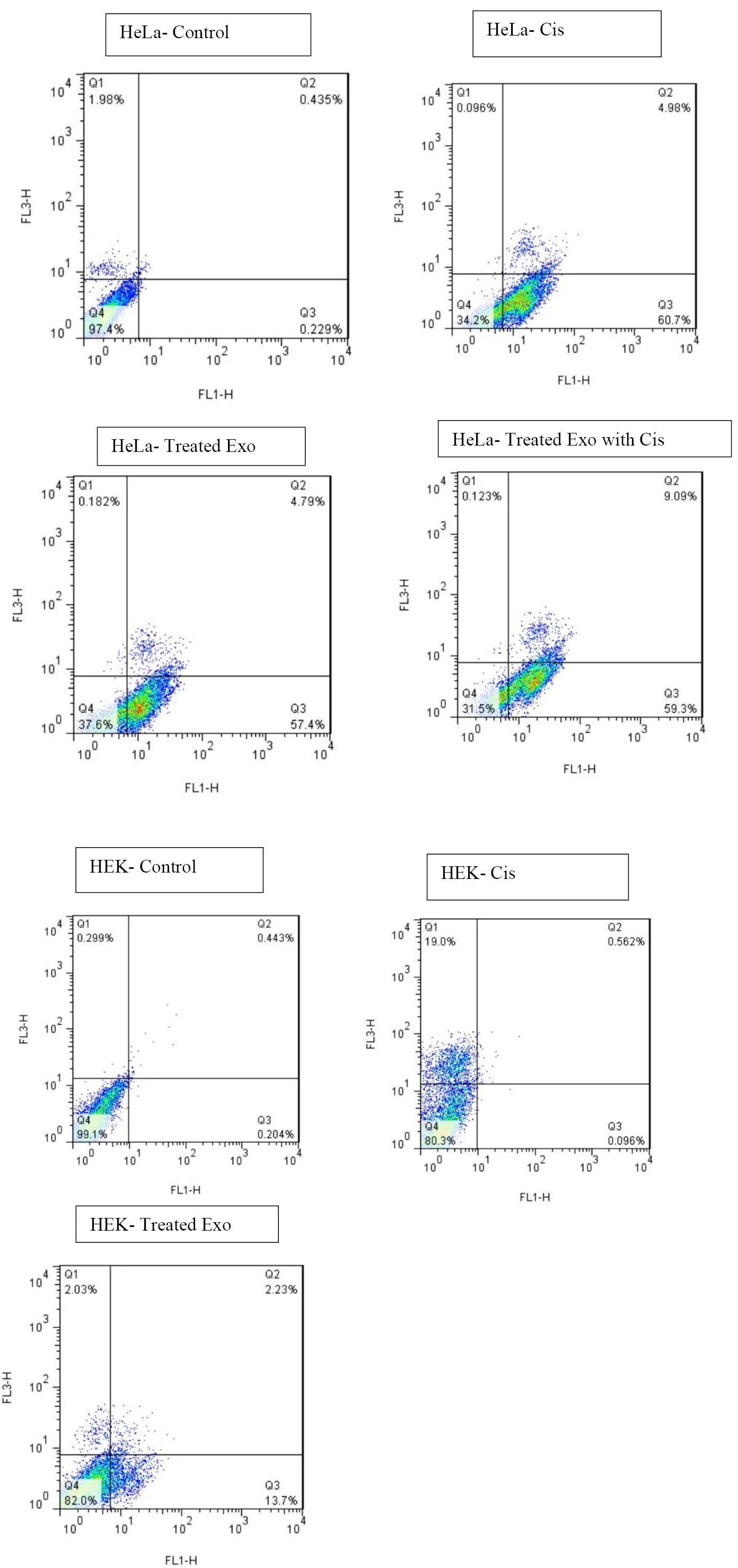
Flow cytometry evaluation of apoptosis. Naïve HeLa/HEK cells were treated with drugs alone, Cis (7.95µM), Exosomes extracted from HeLa cells treated with Cis (7.95µM), Exosomes extracted from naïve HeLa cells (168 μg/ml), Exosomes extracted from HeLa cells treated with Cis (168 μg/ml), and Cis (7.95 µM). The lower left quadrant shows viable cells; the upper left quadrant shows necrotic cells; the lower right quadrant shows early apoptotic cells; the upper right quadrant shows late apoptotic cells

 In recent years, there has been a considerable amount of momentum toward developing cancer treatments that are safer and more effective for the patient’s therapeutic context. Biologically inspired, drug-free therapeutic strategies have received particular interest in this area. The current work presents a novel therapeutic strategy for cervical cancer through the use of exosomes from cisplatin-exposed HeLa cells. The highlight of the current work is that, unlike conventional thinking about exosomes being a vehicle for the delivery of chemotherapeutic drugs, exosomes are potential active players in the treatment.^14-16^ Our results introduce a novel concept, which we term “Effect Delivery.” This perspective emphasizes that exosomes are not merely passive carriers but can actively transmit stress-induced bioactive signals that influence the behavior and destiny of recipient cancer cells.^17,18^ Cervical cancer continues to pose a significant global health challenge, especially in low- and middle-income countries, where it is associated with high levels of both morbidity and mortality.^19-21^ Although cisplatin remains a cornerstone of standard chemotherapy, its clinical utility is often limited by the development of chemoresistance, off-target toxic effects, and the frequent recurrence of disease.^22-24^ The emergence of cisplatin resistance is closely linked to the dysregulation of apoptosis-related genes, deficiencies in DNA damage response mechanisms, and alterations in cellular signaling pathways. These challenges highlight the urgent need to explore alternative therapeutic strategies.^25,26^ Our results show that cisplatin-exposed HeLa cells’ exosomes can activate apoptosis in naïve, unhandled HeLa cells. Crucially, this pro-apoptotic effect showed up even in the absence of measurable cisplatin in the exosomal cargo. These results suggest that the therapeutic activity is mediated by endogenous variables—most probably microRNAs, stress-induced proteins, and other bioactive substances—that are selectively packaged into exosomes in response to chemotherapeutic pressure.^27,28^ Gene expression profiling of recipient HeLa cells demonstrated a pronounced shift toward apoptotic signaling, characterized by the upregulation of BAX, TP53, and Caspase-3, along with the downregulation of BCL2 and BIRC5 (Survivin).^29-31^ The simultaneous increase in TNF-α further implicates the activation of the extrinsic apoptotic pathway and may also reflect an accompanying immunomodulatory or inflammatory response.^32-34^ Flow cytometry investigation verified the pro-apoptotic effect of exosomes extracted from treated cells, resulting in apoptosis values on par with those seen with direct cisplatin therapy. Importantly, non-cancerous HEK293 cells showed little reaction to the same exosomal exposure, implying a tumor-specific vulnerability. This selective action of “effect-loaded” exosomes not only improves their therapeutic index but also suggests a great approach for the creation of directed cancer treatments. Maintaining the anti-cancer signaling effects of “Effect Delivery” via exosomes offers a new dimension of intercellular communication with possible therapeutic benefits. This approach shows great potential for the creation of next-generation, low-toxicity, and individualized cancer treatments by means of chemotherapy while preventing the systemic toxicities often related to drug delivery. Moreover, exosomes’ intrinsic ability to target tumors offers particular benefits in precision medicine as it lets them selectively home to cancer cells and provide highly specific molecular signals.^35-38^ Interestingly, our findings appear to contrast with a substantial body of literature reporting that tumor-derived exosomes, particularly under chemotherapeutic stress, often promote drug resistance, survival signaling, and reduced apoptosis in recipient cells. This discrepancy may reflect the highly context dependent nature of exosome biology. We proposed that the molecular composition and functional impact of exosomes are strongly influenced by the type, intensity, and duration of the inducing stimulus. In the present study, Cisplatin exposure likely triggered a pronounced stress response in donor HeLa cells, resulting in the selective packaging of pro-apoptotic and stress-related molecules, including regulators associated with the TP53 axis, BAX activation, and inflammatory mediators such as TNF-α. This altered exosomal cargo may account for the observed pro-apoptotic effects in recipient cells, in contrast to the resistance-promoting roles reported elsewhere. Our gene expression and apoptosis data consistently support this shift toward apoptotic signaling. These findings suggest that exosomes are not inherently pro-tumorigenic or pro-survival, but rather function as dynamic mediators of intercellular communication whose biological effects can be reprogrammed under specific therapeutic conditions. The observation that exosomes derived from cisplatin-treated cells induced apoptosis in recipient HeLa cells may initially appear inconsistent with previous studies reporting that tumor-derived exosomes contribute to chemoresistance. However, the biological cargo of exosomes strongly depends on the physiological state of the donor cells. When cancer cells are exposed to chemotherapeutic stress, significant alterations occur in their transcriptomic and proteomic profiles, which may subsequently influence the molecular composition of released exosomes.

 Therefore, exosomes secreted by drug-treated cells may contain pro-apoptotic molecules, stress-response proteins, or regulatory RNAs capable of activating apoptotic signaling pathways in neighboring cells. In this context, the apoptotic effect observed in the present study may reflect the transfer of stress-induced signals rather than a classical drug-delivery mechanism. This finding supports the concept that exosomes can function as mediators of intercellular stress communication, potentially amplifying the cytotoxic effects of chemotherapy within the tumor microenvironment. This approach is in line with emerging trends in onco-nanomedicine and exosome engineering, where research increasingly focuses on harnessing cell-derived vesicles as intelligent carriers of therapeutic signals rather than traditional pharmacological drugs.

## Study Limitations and Future Perspectives

 Although the present study provides evidence that exosomes derived from cisplatin-treated HeLa cells can induce apoptotic responses in recipient cervical cancer cells, several limitations should be considered. First, the molecular cargo responsible for the observed apoptotic effects was not specifically identified. Exosomes contain a complex mixture of proteins, microRNAs, mRNAs, and lipids, any of which may contribute to the activation of apoptotic signaling pathways. Therefore, further proteomic and transcriptomic profiling of the isolated exosomes would be necessary to determine the key molecules mediating these effects.

 Second, the current study was performed under in-vitro conditions using established cell lines. While in-vitro models provide valuable mechanistic insights, they cannot fully replicate the complexity of the tumor microenvironment in vivo, where multiple cell types, extracellular matrix components, and immune interactions influence tumor progression and therapeutic responses. Consequently, additional studies using animal models are required to evaluate the therapeutic potential, safety, and biological behavior of these exosomes in more physiologically relevant systems.

 Another limitation is the lack of detailed analysis regarding the mechanisms governing exosome uptake by recipient cells. The efficiency of exosome internalization and the intracellular trafficking of their cargo may significantly influence their biological effects. Understanding these mechanisms could help optimize the therapeutic application of exosome-based strategies and improve their specificity toward cancer cells.

 Future research should therefore focus on identifying the specific bioactive components within exosomes derived from drug-treated cancer cells that are responsible for triggering apoptotic signaling. Advanced approaches such as RNA sequencing, proteomic analysis, and functional inhibition studies could help clarify the molecular pathways involved. In addition, investigating whether similar effects can be observed with exosomes derived from other chemotherapeutic treatments or different cancer cell types would provide valuable insight into the generalizability of this phenomenon.

 Furthermore, engineering or selectively enriching exosomes with pro-apoptotic molecules may represent a promising strategy for developing novel therapeutic approaches. By harnessing the natural intercellular communication system of exosomes, it may be possible to design safer and more targeted anticancer treatments that reduce systemic toxicity while maintaining therapeutic efficacy.

 Overall, the concept of exosome-mediated effect delivery introduced in this study highlights a potentially important mechanism of chemotherapy-associated signaling within the tumor microenvironment. Continued investigation into this process may contribute to the development of innovative therapeutic strategies for cervical cancer and potentially other malignancies.

## Conclusion

 Despite the lack of medicine inside their payload, exosomes obtained from cisplatin-treated HeLa cells can cause apoptosis in untreated HeLa cells. This new phenomenon—Effect Delivery—challenges the established idea of exosomes as inactive drug carriers and emphasizes their inherent capacity to transmit therapeutic signals created by donor cells. Our findings strongly support a drug-free, exosome-mediated approach to getting rid of cancer cells, therefore lowering the overall toxicity linked with standard chemotherapy. Furthermore, emphasizing the potential of this method for focused treatment is the seen selectivity for cancer cells over normal cells. Future research ought to seek to find the main bioactive chemicals in these exosomes and verify the processes causing apoptosis induction in in vivo models, therefore, the means by which Effect Delivery as a new modality in cancer therapy can be translated clinically.

 In summary, this study demonstrates that exosomes derived from cisplatin-treated HeLa cells can induce significant apoptotic responses in recipient cervical cancer cells. The observed increase in pro-apoptotic gene expression, including BAX, Caspase-3, TP53, and TNF-α, together with the reduction of anti-apoptotic markers such as BCL2 and BIRC5, suggests that these exosomes transfer molecular signals capable of activating apoptotic pathways. Importantly, these effects occurred in the absence of direct cisplatin exposure, indicating that drug-induced cellular stress signals can be propagated through exosome-mediated communication.

 These findings support the concept of effect delivery, in which the biological consequences of chemotherapy are transmitted via exosomal signaling molecules rather than the chemotherapeutic agent itself. This mechanism may contribute to amplifying anticancer effects within the tumor microenvironment and could represent a novel strategy for cancer treatment. Further studies aimed at identifying the specific molecular cargo responsible for these effects and evaluating their therapeutic potential in in-vivo models may help advance the development of innovative and less toxic exosome-based therapies for cervical cancer.

## Availability of Supporting Data

 Yes.

## Competing Interests

 The authors declare no relevant financial or non-financial interests.

## Consent for Publication

 Not applicable. This article does not contain any person’s data in any form.

## Ethical Approval

 Not applicable. This article is original and does not involve any studies with human participants or animals performed by any of the authors.

## Human Ethics

 Not applicable. This article is a review and does not report on or involve any studies with human participants conducted by the authors.
